# Negative Affectivity Moderates the Relationship between Attentional Control and Focused Skin Picking

**DOI:** 10.3390/ijerph19116636

**Published:** 2022-05-29

**Authors:** Katarzyna Prochwicz, Rachela Antosz-Rekucka, Alina Kałużna-Wielobób, Dominika Sznajder, Joanna Kłosowska

**Affiliations:** 1 Institute of Psychology, Jagiellonian University, ul. Ingardena 6, 30-060 Kraków, Poland; katarzyna.prochwicz@uj.edu.pl (K.P.); r.antosz-rekucka@doctoral.uj.edu.pl (R.A.-R.); 2 Institute of Psychology, Pedagogical University, ul. Podchorążych 2, 30-084 Kraków, Poland; alina.kaluzna-wielobob@up.krakow.pl (A.K.-W.); dominika.sznajder@up.krakow.pl (D.S.)

**Keywords:** focused skin picking, attentional control, negative affectivity, moderation analysis

## Abstract

Very little is known about the cognitive functioning of people with body-focused repetitive behaviours and the few existing studies provide mixed findings. The aim of this study was to investigate the interplay between attentional control, negative affectivity, and focused skin picking. We hypothesized that the control of attention is associated with focused style of skin picking and that this relationship is moderated by negative affectivity. The final sample consisted of 273 non-clinical subjects (79% women) aged 18 to 54 years; study variables were assessed using questionnaires. Moderation analysis was conducted, followed by a simple slope analysis, and the Johnson–Neyman technique was used to probe the interaction effect. We found that at the low level of negative affectivity, the relationship between attentional control and focused skin picking is negative, but not at the higher levels of this trait. Interestingly, when negative affectivity reaches very high intensity, the association between attentional control and skin picking becomes positive. This relationship seems to be quite complex and may depend on the way that cognitive abilities are used by the individual, as well as on the stage of cognitive processing that they are applied to. Further studies using behavioural measures of attention are needed to better understand this issue.

## 1. Introduction

Skin picking (excoriation) disorder is a mental health condition characterised by repetitive scratching or picking of the skin resulting in skin damage, such as minor sores, hyperpigmentation, shallow scars, and—less frequently—deeper skin disfigurements and skin infections [1,2,3]. Apart from medical consequences, psychological sequelae of skin picking were also identified, including clinically significant distress and functional impairment in various domains [4,5,6]. Skin picking is conceptualised as body-focused repetitive behaviours (BFRBs) due to the presence of recurrent and habitual actions directed at the body (see [7]). BFRBs are currently classified under the broader diagnostic category ‘Obsessive-compulsive and related disorders’ (OCRD) together with other psychiatric conditions which are manifested by repetitive thoughts and/or behaviours. These disorders are also believed to share similar aetiology [7,8].

Although current diagnostic criteria consider skin picking as a homogeneous disorder, empirical studies and clinical observations have provided evidence for its heterogeneity. So far, at least two different skin picking subtypes have been differentiated: automatic skin picking, which is performed habitually and without awareness, and focused skin picking, which is undertaken intentionally [9,10]. The heterogeneity of skin picking raises the question of whether the different picking styles may be associated with different underlying factors. Focused skin picking, similarly to focused hair pulling among individuals with trichotillomania, is considered to be performed to reduce negative internal states (e.g., urges or cognitions) or emotions (e.g., anxiety, depression, boredom) [11], whereas automatic types of BFRBs appear more as a form of self-stimulation [12]. Indeed, existing studies have indicated that automatic and focused skin picking may be differently involved in emotion regulation processes [10,13,14].

It is widely confirmed that people suffering from skin picking experience negative affective states before picking episodes and report relief as a result of picking [14,15,16,17,18]. From this point of view, negative emotions, as well as unpleasant tension that they are usually accompanied with, are considered as picking triggers, whereas skin picking itself is considered as an attempt to reduce given negative states through repetitive activity. The role of skin picking in emotion regulation has received general support in a large body of studies (e.g., [4,6,16,17]). However, a growing number of findings indicate that focused rather than automatic skin picking is involved in emotion regulation [9,10,14,15]. Aware and volitionally controlled picking was confirmed to be preceded by feelings of tension which can be reduced only during the act of picking [14,15] more often than automatic picking, which is performed unconsciously, mostly during sedentary activities [10].

Limited data are available on the neurobiological mechanisms involved in the aetiology of the skin-picking disorder (SPD). However, previous studies on this topic showed impaired performance on the tasks measuring the ability to suppress impulsive and premature motor responses, i.e., the stop signal task (SST) [19], suggesting impaired integrity of neural networks, including the right inferior frontal gyrus and the anterior cingulate cortices (e.g., [20]). Moreover, as has already been pointed out [21], skin picking has a strong motor component, which may implicate additional brain areas involved in the mediation of compulsive–impulsive symptoms [22]. Indeed, white matter abnormalities, including reduced integrity of white matter connecting anterior cingulate cortices [23], a greater volume of the bilateral nucleus accumbens, and reduced cortical thickness in the right frontal areas [24], were found in skin-picking patients in structural neuroimaging studies. Additionally, functional imaging studies showed alternations regarding activation of the basal ganglia, the insula, and the anterior cingulate cortex during executive planning tasks and exposure to affective pictures [14,25,26]. Evidence also exists that SPD patients display structural and functional abnormalities in cerebellum subregions related to motor and affective-cognitive functions [21]. Many neurological impairments manifest themselves as cognitive deficits which can subsequently affect the severity of skin picking, as well as the ability to effectively cope with symptoms.

Although there are a lack of systematic studies on neurocognitive functioning in skin picking, some neurocognitive deficits have also been detected in individuals with BFRBs in neuropsychological examination. Decreased spatial working memory [27], visuo-spatial learning [28], and cognitive flexibility were reported in patients with trichotillomania. One study also yielded deficits in attentional processes [29] measured by neurocognitive tasks (divided attention domain), suggesting that BFRBs may be associated with diminished attentional control, which has been theorized to be central to adaptive emotion regulation [30]. Indeed, studies have shown that cognitive control is associated with more effective use of emotion regulation methods and contributes substantially to adaptive emotion regulation strategies such as cognitive reappraisal [31]. Thus, individuals with a low ability to control attention, as well as lower access to effective strategies, may be more inclined to rely on maladaptive methods of emotion regulation, with skin picking being one of them. This relationship can be visible especially among individuals who have a disposition to experience aversive emotional states, such as those high on negative affectivity [32].

On the other hand, evidence has also been found that individuals suffering from trichotillomania exhibit a tendency to disengage attention from disorder-related cues (hair) and general emotional threat cues at the late stage of attentional processing [33]. This result indirectly supports the notion that individuals with trichotillomania may in fact show relatively good attentional control; however, they do not necessarily use this ability in an adaptive manner. The enhanced disengagement of attention from threat-related stimuli is likely an attempt to reduce negative effects by avoiding upsetting cues. This strategy is potentially counterproductive because attentional avoidance may use up the resources that would otherwise be invested in controlling the symptoms [34]. Previous research has shown that in patients with trichotillomania experiential avoidance, i.e., the tendency to avoid negative internal experiences is associated with hair-pulling symptoms [35] and decreases during psychotherapy along with the changes in disorder severity [36]. Thus, increased ability to voluntarily allocate attention may in fact be associated with a higher level of BFRBs symptoms, especially if excessively invested in down-regulating negative emotions by averting attention away from negative experiences. This relationship is probably more likely to be observed in cases of individuals with a focused type of BFRBs, as constant regulatory efforts aimed at unpleasant emotions are particularly characteristic for them [16,17]. Moreover, individuals high on negative affectivity who tend to experience intense negative emotions all the time [32] may be especially motivated to use their cognitive capacity to avoid unpleasant triggers, whether internal or external. Indirect evidence for this notion comes from studies on neuroticism which have shown that this trait is significantly associated with experiential avoidance [37]. Moreover, research on clinical samples has shown that patients with anxiety disorders present a vigilance–avoidance pattern of cognitive bias; vigilance towards threats in the environment and activation of fear is followed by attentional disengagement, which is probably an attempt to compensate for the early activation of threat response [38].

The aim of the present study was to investigate the relationship between the ability to control attention, negative affectivity, and skin-picking behaviours in a non-clinical sample. Given the fact that BFRBs seem to be associated with numerous neurocognitive impairments [14,21,23,24,25,26] and that low ability to control attention may facilitate the use of maladaptive strategies to cope with negative affect, such us pathological skin manipulation [31], we hypothesized that there would be a negative relationship between attentional control and focused skin picking. Moreover, we hypothesized that negative affectivity can moderate this association in such a way that the link between impaired attentional control and focused skin picking can be stronger in case of individuals who have a tendency to experience frequent and intense negative emotions.

## 2. Materials and Methods

### 2.1. Participants

The study was part of a larger research project. Some of the findings from this project, answering research questions different from the ones currently investigated, have already been described in a previous publication [39]. Initially, 600 participants (76.5% women, 22.8% men, 0.5% non-binary) took part in this study. The inclusion criteria for participants were 18 years of age or older and living currently in Poland. From the initial sample, we excluded participants who declared that they do not pick the skin (*n* = 275), and those who suffered from a dermatological illness (*n* = 52). The final sample consisted of 273 participants (79.1% women, 20.1% men, 0.70% non-binary), aged 18 to 54 years (M = 22.89, SD = 5.62). Among them, 43.6% declared that they were single. Furthermore, 61.17% participants were students, 16.12% were employed, 22.34% reported that they were working and studying at the same time, and 0.37% stated they were unemployed. In addition, 38.10% participants declared they grew up in a city with more than 100,000 inhabitants, 29.30% in smaller cities, and 32.60% in a village. All of the participants identified themselves as white.

### 2.2. Measurements

#### 2.2.1. Attentional Control Scale (ACS)

The Attentional Control Scale is a self-rating scale that consists of 20 items rated on a 4-point Likert scale (from 1 = almost never to 4 = always) which assesses the ability to focus, shift, and divide attention. In our study, we used the Polish version of the ACS and, as recommended by the authors [40], we assumed that the scale is unidimensional and that the sum of scores reflects the general capacity to voluntarily control attention. Cronbach’s alphas calculated for the general ACS score in the current sample was 0.84.

#### 2.2.2. Positive and Negative Affect Schedule (PANAS)

The PANAS is a self-report measure consisting of two scales measuring positive (10 items) and negative effects (10 items). It has two versions measuring current emotional states and constant affective traits. In the current study, the Polish version of PANAS [41] measuring trait affect was utilized. Individuals were asked to rate the extent to which they generally experienced each mood state on a 5-point scale (from 1 = very slightly to 5 = extremely). The scale has very good psychometric properties. In the current sample, Cronbach’s Alphas were 0.91 for both positive affectivity and negative affectivity.

#### 2.2.3. The Milwaukee Inventory for the Dimensions of Adult Skin Picking (MIDAS)

The MIDAS [10] consists of 12 items and assesses skin-picking styles. It contains two subscales measuring automatic (6 items) and focused (6 items) skin picking. The items were rated on a 5-point Likert scale (from 1 = not true of any of my skin picking to 5 = true for all of my skin picking). We used a Polish translation of the MIDAS with the Cronbach’s alphas calculated for the current sample: α = 0.75 for the focused skin picking subscale and α = 0.73 for the automatic skin picking subscale. Since experiences of negative emotional states are particularly involved in focused skin picking [16,17,18], and earlier studies have shown significant association between attentional control and a focused type of BFRBs [33], only the scale measuring focused skin picking was used in the analyses.

#### 2.2.4. Diagnostic Criteria for Skin-Picking Disorder

The participants also answered the four Yes or No questions referring to DSM-5 diagnostic criteria of excoriation (skin-picking) disorder [8]. Those questions were as follows: (1) Do you pick the skin to such an extent that it results in noticeable skin damage?; (2) Have you made attempts to decrease or stop picking?; (3) Do skin-picking behaviours cause clinically significant distress or impairment in social, occupational, or other important areas of functioning; (4) Do you suffer from a psychiatric or dermatological illness which caused picking? Based on their answers, participants were classified into two categories: SPD (skin-picking disorder) absent (*n* = 205) and SPD present (*n* = 68).

#### 2.2.5. Sociodemographic Data Sheet

This datasheet was completed by the participants at the beginning of the survey and included questions about age, gender, race, marital status, employment, and place of origin.

### 2.3. Procedure

The procedure was described in detail in Kłosowska et al.’s [39] article. Data were collected through an online survey conducted between July 2019 and December 2020. The participants were recruited using the convenience sampling method. The link to the survey was disseminated through social media in order to reach as large a number of subjects as possible. At the beginning, participants gave informed consent by checking the appropriate box on the first page of the survey. They were informed that participation is voluntary, the study is anonymous, the data are collected for scientific purposes only, and that they can quit the survey at any point without explaining their reasons for doing so. In the next step, they answered the questions about their gender, age, race, employment, and relationship status. They also indicated if they meet the DSM-5 criteria of skin-picking disorder. In the following step, they completed a series of questionnaires including ACS, PANAS, and MIDAS. The procedure was reviewed and accepted by the Local Ethics Committee.

### 2.4. Data Analysis Plan

In the first step, Pearson correlations between all study variables were examined to check if attentional control, negative affectivity, and skin-picking behaviours were associated. In the case of nominal variables, the phi coefficient was calculated. To verify the hypothesis if the relationship between attentional control and focused skin picking was moderated by negative affectivity, the SPSS PROCESS macro [42] was used. Model 1 (simple moderation) was implemented. The predictor (attentional control), moderator (negative affectivity), and interaction term (attentional control × negative affectivity) were entered simultaneously into the regression analysis, with age, gender, and positive affectivity treated as covariates. Continuous variables were mean-centred before creating interaction terms to improve the interpretability of results. The bias-corrected method with 5000 bootstrap samples was used to assess the 95% confidence intervals of the effects. To probe significant interaction effects, both the pick-a-point technique and the Johnson–Neyman technique were used [43]. For the pick-a-point technique, we examined the relationship of the predictor with the criterion at high (86th percentile), medium (50th percentile), and low (14th percentile) values of the moderator. In the Johnson–Neyman technique, we examined the cut-off scores for the moderation effects. Cases with missing data were excluded from the analyses. Analyses were conducted using SPSS version 27.

## 3. Results

### 3.1. Preliminary Analyses

The results of correlation analysis as well as descriptive statistics are presented in Table 1. Focused skin picking correlated negatively and significantly with attentional control and positive affectivity, and positively with negative affectivity. Not surprisingly, it also correlated positively with absence/presence of SPD. Higher attentional control correlated with increased positive affectivity and decreased negative affectivity.

### 3.2. Moderation Analysis

Moderation analysis showed that the interaction effect of attentional control and negative affectivity on the focused type of skin picking was significant (B = 0.01, SE = 0.004, 95% CI = 0.005 to 0.019, *p* < 0.01, ΔR^2^ = 0.034), indicating that the effect of attentional control on focused skin picking becomes weaker and more positive as negative affectivity increases. The results can be found in Table 2.

**Figure 1:**
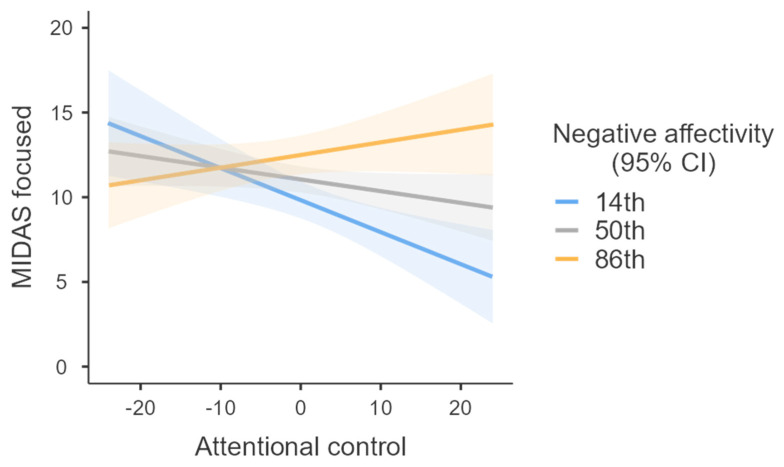
Relationship between attentional control and focused skin picking at different levels of negative affectivity.

To investigate significant interaction effects, we firstly utilized simple slopes analysis [44], and the conditional effects of attentional control on focused skin picking at various levels of negative affectivity were estimated. In each analysis, age, gender, and positive affectivity were treated as covariates. The effect of attentional control on focused skin picking was negative and significant for low levels (14th percentile) of negative affectivity (B = −0.189, SE = 0.058, 95% CI = −0.303 to −0.075, *p* < 0.001), but was insignificant for medium (50th percentile) levels (B = −0.069, SE = 0.039, 95% CI = −0.146 to 0.008, *p* = 0.079) and higher levels (86th percentile) in this trait (B = 0.075, SE = 0.054, 95% CI = −0.031 to 0.180, *p* = 0.164) (Figure 1).

The low level of negative affectivity was fixed at the 14th percentile, medium level at the 50th percentile, and high level at the 86th percentile. The relationship between attentional control and focused skin picking was negative and significant only at the low level of negative affectivity. The slopes did not approach significance at medium and high levels. Gender, age, and positive affectivity were controlled for. Additional analyses conducted separately for women and men can be found in the Supplementary Materials (Figures S4–S7).

**Figure 2:**
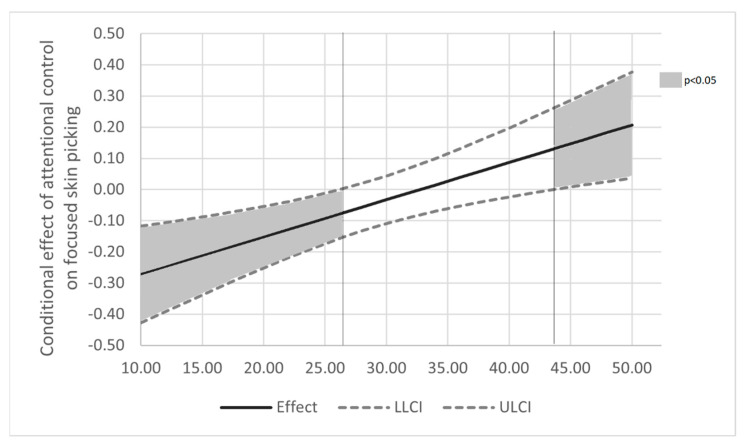
The Johnson–Neyman graph for the model relating focused skin picking to attentional control, negative affectivity and their interaction. Note: The effect of attentional control on focused skin picking is negative and significant for lower levels of negative affectivity (<26.25) and positive and significant at high levels of negative affectivity (>43.71). Gender, age and positive affectivity were controlled for in the analysis. Additional analyses conducted separately for women and men can be found in Supplementary Materials (Figures S4–S7).

Since values for low, medium, and high levels of the moderator variable are arbitrary in simple slope analysis, and as this method does not provide a high-resolution picture of precisely at which point a level of negative affectivity makes a difference in determining whether attentional control is associated with focused skin picking, we applied the Johnson–Neyman (J-N) technique to further probe the interaction effect [43]. This method examines at what level(s) of the moderator the conditional effect of predictor on the dependent variable is statistically significant. Figure 2 presents the Johnson–Neyman graph for the effect. Interestingly, J-N showed that the effect of attentional control on focused skin picking is significantly different than “0” for values of negative affectivity lower than 26 (negative effect), but also for the values higher than 43 (positive effect). It indicates that for lower negative affectivity, higher attentional control is associated with a lower level of focused skin picking; for medium and elevated levels of negative affectivity, the relationship between attentional control and skin picking is insignificant; and when negative affectivity reaches very high levels (>mean + 2SD), attentional control becomes in fact significantly and positively associated with focused skin picking. Results of the J-N procedure are therefore consistent with the simple slope analysis, at the same time offering a more detailed picture of the interplay between attentional control, negative affectivity, and skin-picking behaviours.

## 4. Discussion

The study confirmed our hypothesis that a lower ability to control attention is associated with focused skin picking. Additionally, in line with our prediction, the tendency to experience negative states (negative affectivity) moderated this relationship. However, the direction of interaction effect was different than hypothesized: in the case of individuals with low negative affectivity, attentional control was negatively associated with skin picking; however, among individuals with higher levels of this trait, “beneficial” effect of increased attentional control was no longer visible.

At the initial step of our analyses, we investigated correlational relationships between study variables in order to establish whether attentional control, affectivity, and skin-picking behaviours were associated. First of all, we observed a positive relationship between focused skin picking and negative affectivity, indicating that individuals with a high level of this trait who are prone to intense and negative emotions are more likely to pick at the skin consciously. In general, this finding is in line with numerous studies showing the link between focused skin picking and unpleasant emotional experiences [10,13,14,15,18], and may suggest that episodes of focused picking are triggered by emotion-related tension and aimed at reducing frequent negative internal states [13,14]. It is also worth mentioning that correlational analysis yielded a statistically significant association between gender and focused skin picking, indicating that women engage in focused skin picking more frequently than men. Moreover, in the current sample, women met the diagnostic criteria of skin-picking disorders more frequently than men. Similar findings were obtained in the previous studies [45].

The correlational analyses also revealed that attentional control and focused skin picking are negatively correlated. At first glance, this result may suggest that the significant ability to control attention constitutes a protective factor against the development of skin-picking behaviours, similar to the case of obsessive–compulsive disorder [46]. It is also in line with the studies showing attention deficits in trichotillomania [29]. However, the results of further analyses revealed that this relationship is a little bit more intricate than it first appears.

Moderation analysis yielded the significant interaction effect of attentional control and negative affectivity on focused skin picking. Consistent with correlational data, it was shown that the significant ability to voluntarily control attention is negatively associated with skin picking, however only in conditions where the tendency to experience intensive negative states is relatively weak. For higher levels of negative affectivity, this relationship is no longer significant. Surprisingly, we also obtained results suggesting that when negative affectivity is subjectively rated as very intense, attentional control shows positive association with focused skin picking. Although counterintuitive, the findings obtained are in fact consistent with results of Lee et al.’s [33] study concerning attentional control in trichotillomania. Lee et al. [33] provided evidence that the tendency to allocate attention away from the experiences of negative affect is positively associated with hair-pulling severity. Similarly to what was observed in the current research, in Lee et al.’s study [33], enhanced attention control was linked with the focused style of hair-pulling.

Given that the significant ability to voluntarily control attention can limit access to threatening stimuli that trigger negative affective states [47,48], and at the same time give access to effective emotion regulation strategies [31], it should be associated with reduction rather than exaggeration of behaviours typically related to negative effects such as skin picking. Therefore, it may seem confusing that when negative emotions tend to be particularly intense, the significant ability to control attention, i.e., divert attention away from external or internal threat, does not help or may even be associated with exaggeration rather than a reduction in skin-picking symptoms. As stated previously [33], one possible explanation is that the act of redirecting attention captures cognitive resources which, therefore, cannot be utilized to control skin-picking behaviours. Individuals who tend to experience intense negative emotions can be especially prone to use their cognitive abilities in such a manner, and the depletion of attentional resources makes it especially difficult for them to hold back on skin picking.

It is also possible that high levels of attentional control somehow prevent or handicap the habituation to threat-related cues which are considered as skin-picking triggers. Some studies [38,49] suggest that information processing in patients suffering from disorders characterized by frequent and intense negative affects is characterized by the early attentional selection of emotional stimuli, followed by full conscious awareness, and finally attentional avoidance of threat. It is maladaptive because excessive avoidance, and thus absence of conscious processing, inhibits the extinction that occurs with repeated exposure to threatening cues [50]. Lee et al.’s [33] study suggests that such a pattern may also be characteristic for people suffering from body-focused repetitive behaviours who tend to redirect their attention from threatening cues at the late stage of stimuli processing. It is possible that, due to a lack of habituation, the number of hair-pulling or skin-picking triggers does not diminish over time; therefore, the number of episodes does not decrease. Unfortunately, since we utilized a unidimensional self-report measure of attentional control in the current study [40], we are not able to specify which stage of threat processing is associated with increased skin picking. More research is needed to determine whether the pattern of attention disengagement observed in individuals with trichotillomania occurs in skin-picking sufferers. It also needs to be stressed that positive association between attentional control and skin-picking behaviours was observed in only 4% of participants who obtained more than 43 points on PANAS in our study. Therefore, this finding needs to be treated with caution, and further studies involving bigger samples of participants high in negative affectivity need to be conducted to check if higher attentional control may be disadvantageous for them.

The present study has some other limitations that should be considered when interpreting the results. The study sample consisted mostly of younger women, and the results of additional analyses (see Supplementary Materials Figures S4–S7) suggest that these findings may not generalize to samples consisting primarily of men. Moreover, skin-picking assessment was based solely on self-reports. Future studies could benefit from broadening the range of methods for skin-picking evaluation, e.g., by employing medical examination or face-to-face diagnostic interviews. Additionally, we focused on attentional control as a unidimensional factor, as suggested by the validation study of the instrument [40]. Future studies (for example involving behavioural measures of attentional control) should determine which aspect of attentional functioning is associated with skin picking and negative affectivity. Furthermore, causal relationships between factors cannot be established due to the cross-sectional study design; therefore, experimental and longitudinal studies should be carried out.

## 5. Conclusions

Despite some limitations, this study provides preliminary evidence on the association between attentional control and skin-picking behaviours. It also partially explains previous mixed findings obtained in the studies focusing on trichotillomania [19,20] and concerning attentional efficiency. We demonstrated that the relationship between attentional control and focused skin picking is moderated by negative affectivity. However, in the case of people who do not tend to experience intense negative emotions, better attentional control is related to fewer skin-picking behaviours, which is not necessarily the case for people characterized by high negative affectivity. Among these individuals, the beneficial role of enhanced attentional abilities is not so apparent. They may be motivated to use cognitive abilities to direct attention away from unpleasant negative experiences, thus depleting cognitive resources needed to control their symptoms, and even inhibiting the process of extinction of behaviour. The results of the current study also tentatively suggest that, in the case of individuals with focused skin picking, possible attentional control training should probably be accompanied by emotion regulation training and interventions aimed at reducing experiential avoidance. Considering the potential clinical implications, further studies using experimental paradigms which allow the discrimination of different components and phases of attentional processing are needed to better understand the specific nature of these associations.

## Figures and Tables

**Table 1:** Descriptive statistics and results of correlational analysis.

Variable	Mean (SD)	Min/Max	Kurtosis	Skewness	(1)	(2)	(3)	(4)	(5)	(6)
MIDAS focused (1)	11.630 (5.546)	0/24	−0.647	−0.069	1.000					
PANAS positive (2)	28.092 (8.289)	10/49	−0.609	0.088	−0.232 ***	1.000				
PANAS negative (3)	27.744 (9.310)	10/50	−0.813	0.179	0.270 ***	−0.397 ***	1.000			
ACS total (4)	47.110 (8.839)	24/71	−0.258	−0.019	−0.180 **	0.389 ***	−0.307 ***	1.000		
Age (5)	22.890 (5.624)	18/54	10.276	2.952	−0.122 *	0.075	−0.089	0.004	1.000	
Gender ^a^ (6)	-	-	-	-	0.219 ***	−0.098	0.051	−0.037	−0.071	
Absence/presence of SPD ^b^	-	-	-	-	0.231 ***	−0.018	0.017	−0.082	−0.085	0.14 *^c^

Note: * *p* < 0.05, ** *p* < 0.01, *** *p* < 0.001; *n* = 273 (*n* = 271 for correlations between gender and other variables); SPD—skin-picking disorder; MIDAS—The Milwaukee Inventory for the Dimensions of Adult Skin Picking; PANAS—the Positive and Negative Affect Scale; ACS—the Attentional Control Scale. ^a^ Because only two of the participants indicated “non-binary” gender, only the results for men (coded as 0) vs. women (coded as 1) are presented in the table; ^b^ absence of skin-picking disorders (as determined by the DSM-5 diagnostic criteria) was coded as 0 and the presence of skin-picking disorders was coded as 1; ^c^ phi coefficient.

**Table 2:** Results of moderation analysis.

			Bootstrap (*n* = 5000) 95% CI					
Variable	B	SE	Lower	Upper	t	β	*p*	η2p
Intercept	11.127	0.322	11.287	12.555	3.010	-	<0.001	-
Attentional control	−0.061	0.039	−0.138	0.015	−1.58	−0.100	0.115	0.009
Negative Affectivity	0.121	0.037	0.048	0.194	3.27	0.202	<0.001	0.039
Attentional Control × Negative affectivity	0.012	0.004	0.005	0.019	3.29	0.177	<0.001	0.039
Positive affectivity	−0.064	0.043	−0.149	0.021	−1.48	−0.100	0.140	0.008
Gender ^c^	2.675	0.774	1.150	4.199	3.45	0.194	<0.001	0.043
Age	−0.082	0.055	−0.191	0.027	−1.48	−0.083	0.139	0.008

Note: *n* = 271; F(6, 264) = 9.570, *p* < 0.001, R^2^ = 0.179. Predicted value of MIDAS focused = 11.13 − 0.06 (attentional control) + 0.12 (negative affectivity) + 0.01 (interaction term) − 0.06 (positive affectivity) − 0.08 (age) + 2.68 (gender); ^c^ men were coded as 0 and women were coded as 1.

## Data Availability

The data presented in this study are available upon request from the corresponding author.

## Supplementary Materials

The following supporting information can be downloaded at: https://www.mdpi.com/article/10.3390/ijerph19116636/s1. Figure S1: Results of simple slope analysis; Figure S2: Q-Q plot; Figure S3: Residuals plots; Figure S4: Relationship between attentional control and focused skin-picking at different levels of negative affectivity-women; Figure S5: Relationship between attentional control and focused skin-picking at different levels of negative affectivity-men; Figure S6: The Johnson-Neyman graph for the model relating focused skin-picking to attentional control, negative affectivity and their interaction-women. Figure S7: The Johnson-Neyman graph for the model relating focused skin-picking to attentional control, negative affectivity and their interaction-men. Table S1: Results of moderation analysis; Table S2: Regions of significance—results of Johnson-Neyman procedure; Table S3: Results of autocorrelation test; Table S4: Multicollinearity statistics.
