# Supplementary material for: Negative Affectivity Moderates the Relationship between Attentional Control and Focused Skin Picking
Source: Int J Environ Res Public Health. 2022 May 29;19(11):6636. doi: 10.3390/ijerph19116636 (PMC9180320; doi:10.3390/ijerph19116636)
Supplement: Supplementary file 1 [file ijerph-19-06636-s001.zip › ijerph-1700589-supplementary.pdf]

Supplementary materials

Part A. Attentional control and trait-affect as predictors of skin picking disorder (SPD) – secondary analyses

Table S1. Results of moderation analysis.

			95% CI				
**Variable**	**Estimate**	**SE**	**Lower**	**Upper**	**z**	**Odds ratio**	**p**
Intercept	-0.08	0.15	-1.38	-0.79	-7.15	0.34	<0.001
Attentional control	-0.03	0.02	-0.07	0.01	-1.65	0.97	0.10
Negative affectivity	0.00	0.02	-0.03	0.04	0.10	1.00	0.92
Attentional control x negative affectivity	0.00	0.00	0.00	0.01	2.23	1.00	<0.05
Positive affectivity	0.01	0.02	-0.03	0.05	0.58	1.01	0.56
Gender	0.99	0.44	0.18	1.93	2.24	2.68	<0.05
Age	-0.04	0.03	-0.10	0.02	0.97	-1.17	0.24

Note: dependent variable: presence(1)/absence(0) of skin picking disorder according to DSM-5 diagnostic criteria; men were coded as 0, women were coded as 1

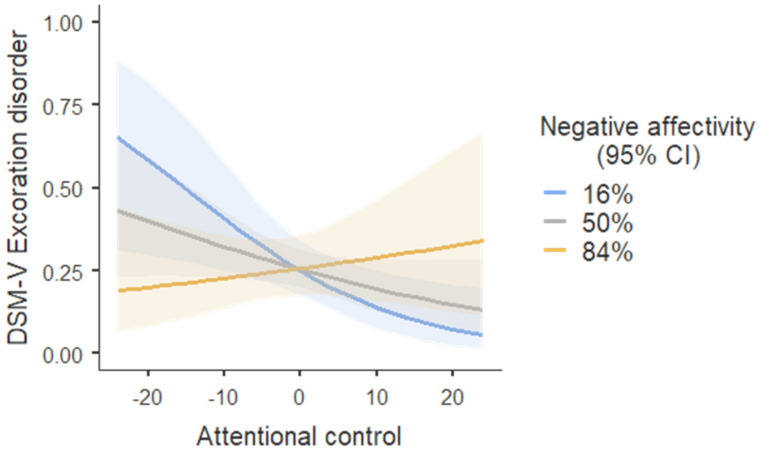

Figure S1. Results of simple slope analysis.

Table S2. Regions of significance – results of Johnson-Neyman procedure.

					95%CI	
**Negative affectivity**	**Effect**	**SE**	**z**	**p**	**Lower**	**Upper**
10	-0.1056	0.0411	-20.5713	0.0101	-0.1862	-0.0251
12	-0.0972	0.0377	-20.5754	0.0100	-0.1711	-0.0232
14	-0.0887	0.0345	-20.5734	0.0101	-0.1563	-0.0211
16	-0.0802	0.0313	20.5611	0.0104	-0.1416	-0.0188
18	-0.0718	0.0283	-20.5317	0.0114	-0.1273	-0.0162
20	-0.0633	0.0256	-20.4742	0.0134	-0.1134	-0.0132
22	-0.0548	0.0231	-20.3720	0.0177	-0.1001	-0.0095

24	-0.0463	0.0210	-20.2029	0.0276	-0.0876	-0.0051
26	-0.0379	0.0195	-10.9430	0.0520	-0.0761	0.0003
28	-0.0294	0.0186	-10.5803	0.1140	-0.0659	0.0071
30	-0.0209	0.0185	-10.1331	0.2572	-0.0571	0.0153
32	-0.0125	0.0191	-0.6521	0.5143	-0.0499	0.0250
34	-0.0040	0.0204	-0.1951	0.8453	-0.0440	0.0361
36	0.0045	0.0223	0.2007	0.8409	-0.0393	0.0483
38	0.0130	0.0247	0.5248	0.5997	-0.0354	0.0613
40	0.0214	0.0274	0.7831	0.4335	-0.0322	0.0751
42	0.0299	0.0303	0.9876	0.3234	-0.0294	0.0892
44	0.0384	0.0334	10.1499	0.2502	-0.0270	0.1038
46	0.0468	0.0366	10.2801	0.2005	-0.0249	0.1186
48	0.0553	0.0399	10.3857	0.1658	-0.0229	0.1336
50	0.0638	0.0433	10.4724	0.1409	-0.0211	0.1487

Part B. Results of assumptions testing for moderation analysis presented in the manuscript

Table S3. Results of autocorrelation test.

Autocorrelation	Durbin-Watson statistic	p
-0.07	2.14	0.31

Q-Q Plot

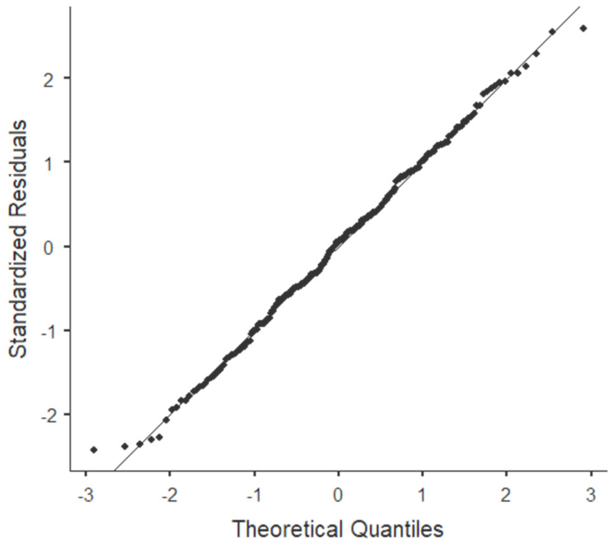

Figure S2. Q-Q plot.

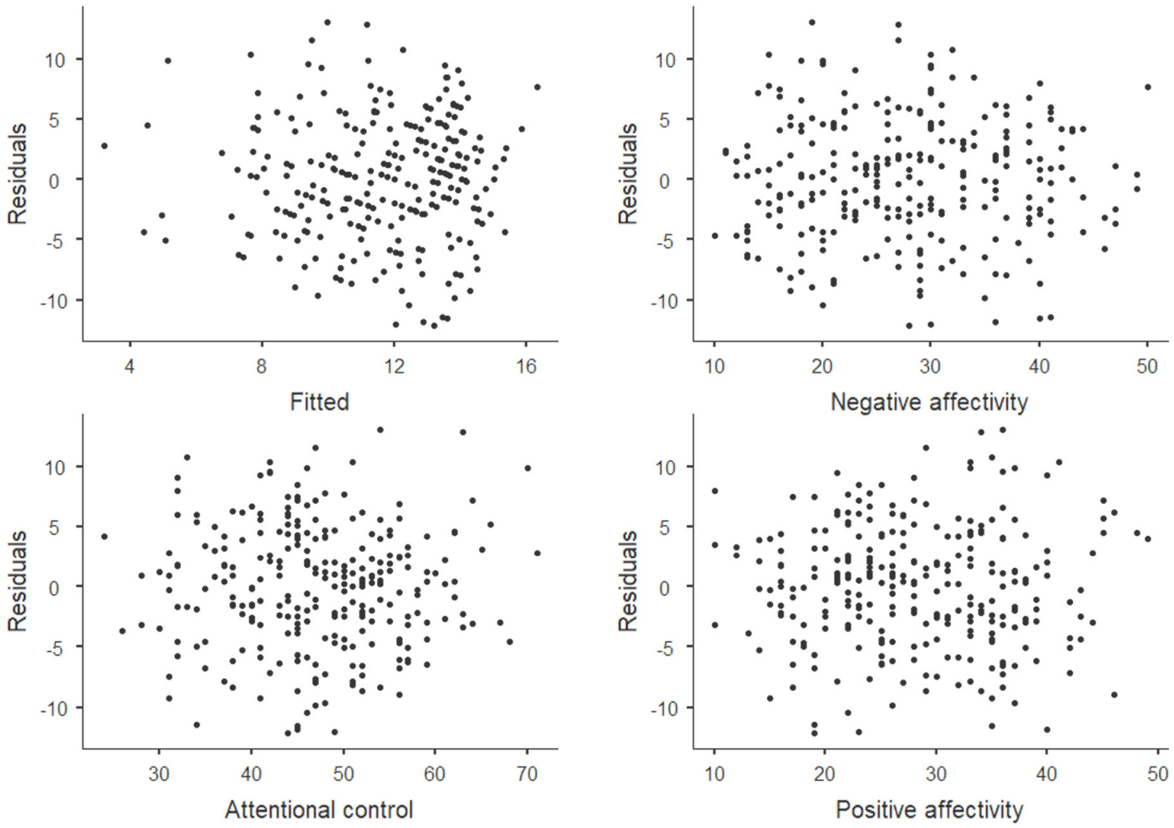

Note: Test of homogeneity of residual variance: F(1,269)=0.05, p=0.82

Figure S3. Residuals plots.

Table S4. Multicollinearity statistics.

Variable	Tolerance	VIF
Attentional control	0.81	1.23
Negative affectivity	0.82	1.22
Attentional control x Negative affectivity	0.98	1.02
Positive affectivity	0.76	1.32
Age	0.99	1.01
Gender	0.99	1.02

Note: Continuous variables were mean-centered before creating an interaction term

Part C. Results of simple slope analysis and J-N method conducted separately for men and women

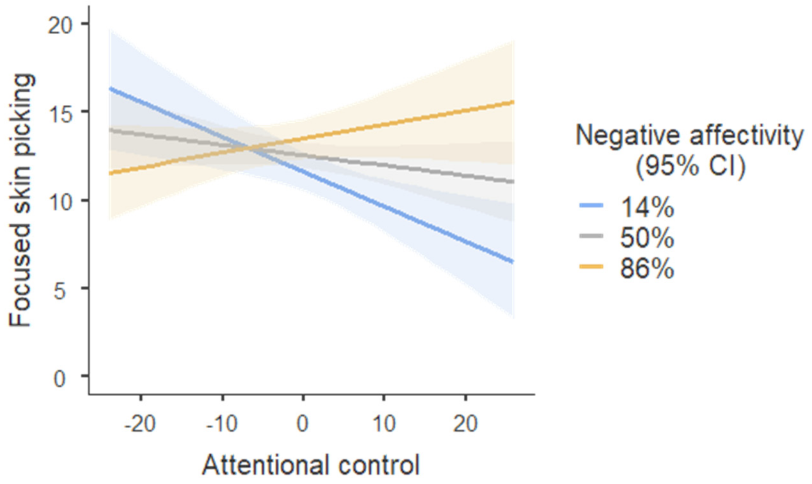

Note: Low level of negative affectivity was fixed at 14th percentile, medium level at 50th percentile, and high level at 86th percentile. The relationship between attentional control and focused skin picking was negative and significant only at low level of negative affectivity. The slopes did not approach significance at medium and high levels.

Figure S4. Relationship between attentional control and focused skin-picking at different levels of negative affectivity - women.

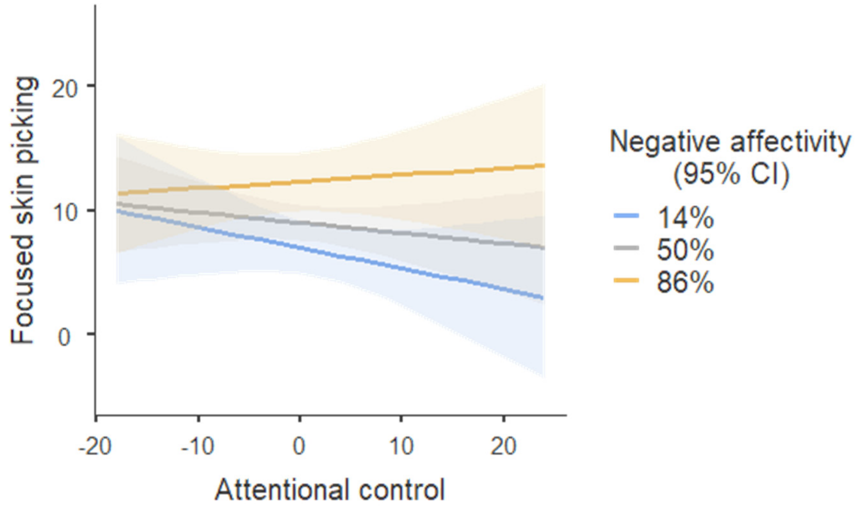

Note: Low level of negative affectivity was fixed at 14th percentile, medium level at 50th percentile, and high level at 86th percentile. The relationship between attentional control and focused skin picking is insignificant at all levels of negative affectivity.

Figure S5. Relationship between attentional control and focused skin-picking at different levels of negative affectivity - men.

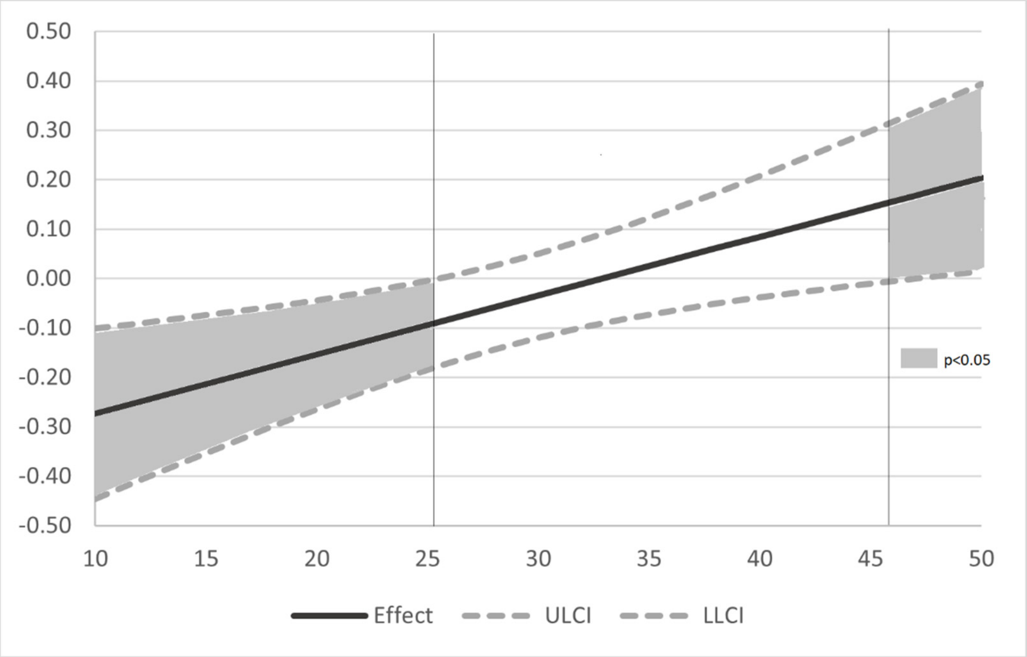

Note: The effect of attentional control on focused skin-picking is negative and significant for lower levels of negative affectivity (<25) and positive and significant at high levels of negative affectivity (> 46).

Figure S6. The Johnson-Neyman graph for the model relating focused skin-picking to attentional control, negative affectivity and their interaction - women.

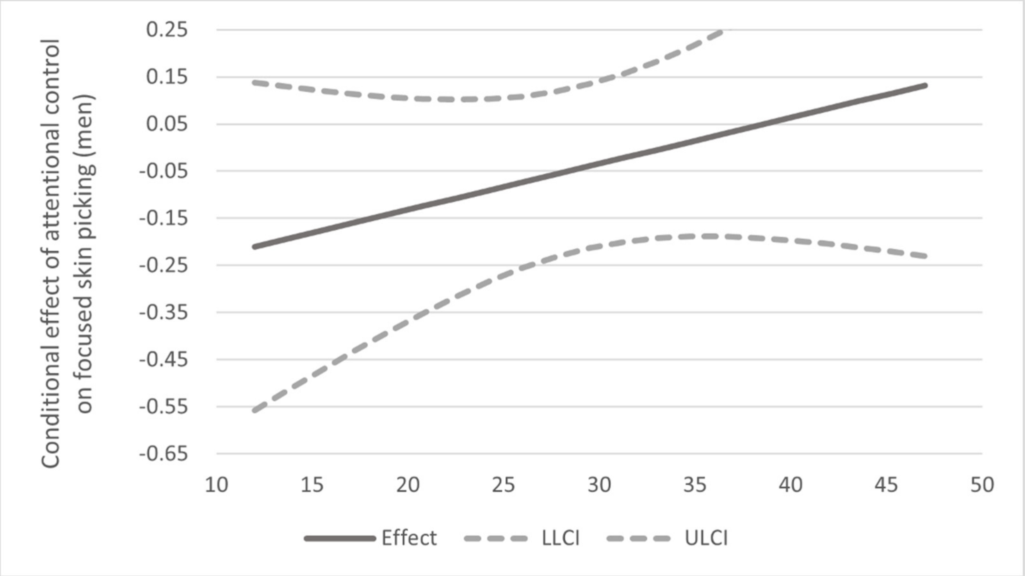

Note: There are no statistical significance transition points within the observed range of the moderator found using the Johnson-Neyman method.

Figure S7. The Johnson-Neyman graph for the model relating focused skin-picking to attentional control, negative affectivity and their interaction - men.
